# Bite to Brain: Unwitnessed Pediatric Neurotoxic Envenomation Mimicking Brain Death

**DOI:** 10.7759/cureus.99916

**Published:** 2025-12-23

**Authors:** Pawan K Ghanghoriya, Shaunak Rangarh, Jayas Jagan, Monica Lazarus, Arvinder Wander

**Affiliations:** 1 Department of Paediatrics, Netaji Subhash Chandra Bose Medical College, Jabalpur, IND; 2 Department of Paediatrics, All India Institute of Medical Sciences, Bathinda, IND

**Keywords:** brain death mimicker, early morning neuroparalytic syndrome, krait bite, neurotoxic envenomation, unwitnessed snake bite

## Abstract

Snakebite envenomation is an important yet treatable cause of mortality. When the bite is witnessed, clinical diagnosis is straightforward, enabling prompt administration of anti-snake venom (ASV). However, in the absence of a witnessed event or local bite-site signs, diagnosing snakebite becomes challenging for emergency physicians. Neurotoxic envenomation, particularly from krait species, can closely mimic acute flaccid paralysis (AFP), often leading to diagnostic uncertainty. We report the case of an adolescent girl who presented with AFP and encephalopathy.

A 13-year-old girl from a rural area presented in the early morning during the monsoon season with acute flaccid paralysis, coma, and signs of brainstem dysfunction (dilated and fixed pupils, absent doll’s eye, and gag reflex). After excluding alternative diagnoses and identifying subtle bite marks, a probable krait bite was considered. The patient made a complete recovery following timely ASV administration, 22 days of mechanical ventilation, and supportive care.

Krait bite should be considered in previously healthy children who present with early morning neuroparalytic syndrome (EMNS) or a brain-death-like clinical picture during the monsoon season.

## Introduction

According to WHO estimates, around 5.4 million people are bitten by snakes each year. Of these, 1.8-2.7 million result in envenomation, leading to 81,410-137,880 deaths annually [1]. In India alone, approximately 1.11-1.77 million snakebites occur each year, causing an estimated 58,000 deaths, nearly 25% of which occur in children [2]. Children have a higher case-fatality rate because their lower body mass results in a higher venom dose per kilogram. The “big four” medically important snakes in India are Russell’s viper, saw-scaled viper, common krait, and cobra, with humped-nosed pit viper bites also common in southern India. Snake venom can produce both local effects (pain, swelling, tissue necrosis) and systemic complications, including hemotoxicity, neurotoxicity, acute kidney injury, and myotoxicity. Snakebite is usually identified by a witnessed bite, characteristic local signs (bite marks, pain, swelling), and classical neurological manifestations such as ptosis, ophthalmoplegia, the broken-neck sign, early morning neuroparalytic syndrome (EMNS), respiratory paralysis, and altered consciousness. Hemorrhagic features may also occur, including hematuria, hematemesis, conjunctival bleeding, gum bleeding, intracranial hemorrhage, and shock [3]. Bite-to-needle time is the most important prognostic factor in snakebite envenomation, especially in neurotoxic bites where clinical deterioration can be rapid. In witnessed bites, diagnosis is usually straightforward, allowing prompt administration of anti-snake venom (ASV). Unwitnessed snakebites, however, are common, reported in 4% to 30% of cases, and present a diagnostic challenge, particularly in young children and when local signs such as bite marks, swelling, or necrosis are absent [3-7]. Diagnosing an unwitnessed krait (*Bungarus caeruleus*) bite is especially difficult. Kraits are nocturnal, have small short fangs, and often inflict painless bites with only mild tingling or numbness. Fang marks may be subtle or absent, and local reactions are minimal or nonexistent [8]. A high index of suspicion is therefore essential. Clues such as a hyperacute presentation, subtle bite marks, EMNS, the broken-neck sign, autonomic features, and timely empirical administration of ASV can be lifesaving in unwitnessed snakebite cases [9,10]. We report the case of a 13-year-old girl who developed quadriplegia, coma, and fixed dilated pupils due to an unwitnessed snakebite.

## Case presentation

A 13-year-old girl from a rural family presented with sudden breathing difficulty and decreased responsiveness. She had been sleeping on the floor at night and developed sudden abdominal pain and vomiting at around 5:00 AM. This was followed by rapidly progressive generalized weakness, respiratory difficulty, and altered sensorium. There was no history of seizures, fever, diarrhea, drug or toxin exposure, trauma, polyuria, polydipsia, weight loss, or any other significant prior illness.

She arrived at the casualty at 7:00 AM. On examination, she was comatose (Glasgow Coma Scale (GCS) E1V1M1), in hypotensive shock (blood pressure 60/40 mmHg), and in respiratory failure (respiratory rate 54/min with poor effort; SpO₂ 86% without oxygen/88% with oxygen). Her temperature was 99°F. She had bilaterally dilated and fixed pupils, absent doll’s eye reflex, generalized hypotonia, quadriplegia, and absent tracheal, deep tendon, and superficial reflexes. Skin and systemic examinations were unremarkable. Random blood sugar (RBS) was 352 mg/dL, and urinary ketones were negative.

Investigations

Laboratory investigations showed normal complete blood counts, HbA1c, serum electrolytes, liver function tests, and renal parameters. Magnetic resonance imaging (MRI) of the brain with whole-spine screening and cerebrospinal fluid (CSF) analysis were also unremarkable (Table 1).

**Table 1:** Laboratory investigations ALT: alanine transaminase, ALP: alkaline phosphatase, AST: aspartate aminotransferase, APTT: activated partial thromboplastin time, HDL: high-density lipoprotein, LDL: low-density lipoprotein, PT: prothrombin time.

Laboratory investigations	Day 1	Normal range
Hemoglobin (g/dl)	11.7	12-15.6
Total WBC count (µL)	4100	3.5-10
Packed cell volume (%)	26.9	30-44
Mean corpuscular volume (fL)	72.4	80-96
Mean corpuscular hemoglobin (pg)	23.7	27-31
Platelet count (µL)	154,000	150,000-450,000
Blood urea (mg/dL)	69.59	7.0-20
Serum creatinine (mg/dL)	0.7	0.6-1.5
Total bilirubin (mg/dL)	0.29	0.1-1.2
Direct bilirubin (mg/dL)	0.05	<0.3
Total protein (g/dL)	6.5	6-8.3
Albumin (mg/dL)	3.05	3.4-5.4
Uric acid (mg/dL)	5.6	2.5-6.8
AST (IU/L)	38.2	0-46
ALT (IU/L)	14	0-49
ALP (IU/L)	187.2	<350
Globulin (g/dL)	2.2	2-3.5
Triglycerides (mg/dL)	42.64	0-150
Total cholesterol (mg/dL)	121.07	40-200
HDL cholesterol (mg/dL)	31.25	35.3-79.5
LDL cholesterol (mg/dL)	81	0-130
Serum sodium (mEq/L)	138.3	135-145
Serum potassium (mEq/L)	4.17	3.4-4.7
Serum chloride (mEq/L)	100.7	98-106
Serum calcium (mg/dL)	9.2	9.0-11
HbA1c (%)	4.8	<5.7
Urine ketone/sugar	Negative	Negative
PT (seconds)	15	10-16
APTT (seconds)	26	22-35

Differential diagnosis

A syndromic diagnosis of acute flaccid paralysis with encephalopathy was made. The differential diagnoses considered included acute meningoencephalitis, Bickerstaff overlap syndrome variant of Guillain-Barré syndrome (GBS), acute demyelinating disorders, snakebite, acute intermittent porphyria (AIP), diabetic ketoacidosis (DKA), stroke, metabolic encephalopathy, and unknown poisoning.

Treatment and outcome

The patient was placed on mechanical ventilation and started on broad-spectrum antibiotics, inotropes, and supportive therapy. Hypertonic saline (3% NaCl) was initiated for suspected raised intracranial pressure, given her comatose state and bilaterally dilated, fixed pupils. An insulin infusion (0.1 IU/kg/hr) was started due to concern for DKA in the context of elevated blood glucose. There was no clinical improvement on day 2 of admission. With negative urinary ketones, normal HbA1c, and normalization of blood glucose, DKA was ruled out, and the insulin infusion was discontinued. A normal MRI brain, CSF analysis, and other laboratory results excluded inflammatory pathologies such as demyelinating disorders and acute meningoencephalitis. GBS, snakebite, and toxin ingestion were therefore strongly considered. The rapid progression from symptom onset to nadir (approximately two hours) and preserved awareness early in the course were atypical for GBS, shifting the focus toward snakebite and toxic exposure. The parents denied the presence of toxins or medications at home and reported that the child had gone to the outdoor washroom during the night. Careful skin examination revealed subtle bite marks on the left foot without local reaction, raising suspicion for krait envenomation. A total of 30 vials of ASV were administered in three aliquots of 10 vials every six hours on day 2 of admission. Neostigmine, pyridostigmine, and calcium infusion were also tried, but without clinical response.

**Figure 1:**
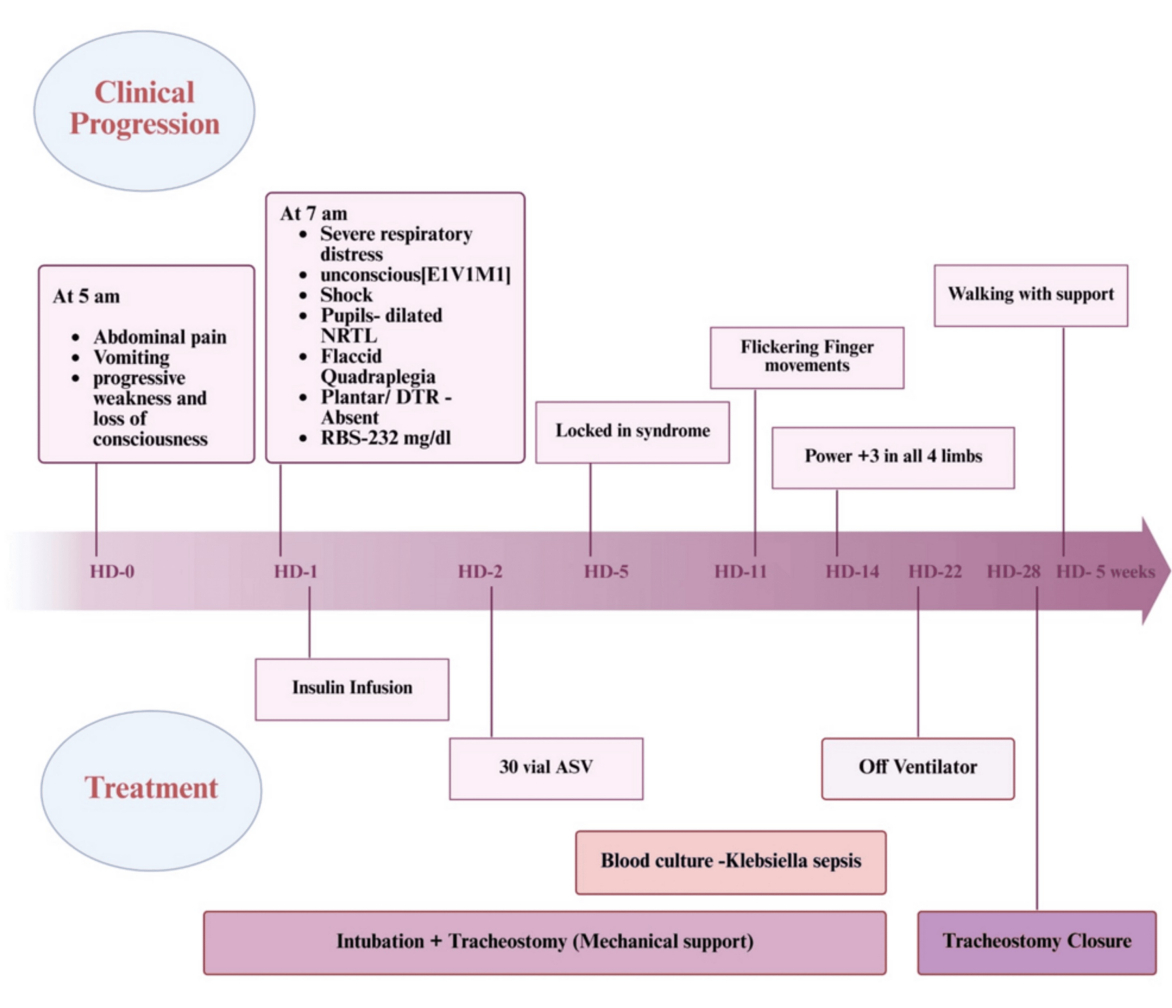
Timeline of the clinical course and management of the patient NRTL: non-reactive to light, DTR: deep tendon reflexes, RBS: random blood sugar, HD: day in hospital, ASV: anti-snake venom.

The patient began to show improvement on day 5, initially with eye opening and communication by blinking, though she remained in a locked-in state with no voluntary limb or eye movements. She underwent tracheostomy on day 10. Flickering finger movements appeared on day 11, and by day 14, her GCS had improved to E4VTM2. Gradual improvement continued: she was weaned off the ventilator on day 22, and the tracheostomy was closed on day 28 (Figure 1). By the end of five weeks, she was able to take a few assisted steps and was discharged. At the two-week follow-up, she was able to walk independently, and her nerve conduction study was normal. At three months, she had regained normal muscle strength in all muscle groups.

## Discussion

Unwitnessed snakebite poses a significant diagnostic challenge for emergency physicians, as the diagnosis is frequently delayed or missed. Misdiagnosis often occurs due to the absence of bite marks and local reaction, unprovoked nocturnal bites during sleep, rapid progression of symptoms leading to paralysis with or without coma, and autonomic manifestations, all of which may initially suggest neurological disorders or poisoning. Unwitnessed bites from cobras and vipers can still be suspected because they usually produce obvious local reactions and fang marks. In contrast, krait bites are particularly difficult to identify, as they occur during sleep, are painless, and typically lack local reaction (due to the absence of cytotoxins in the venom), with bite marks either absent or very faint. Neurotoxic envenomation may result from cobra, common krait, coral snake, or Russell’s viper bites. Cobra and Russell’s viper bites usually cause local tissue reaction, whereas krait and sea snake bites do not. Hemotoxicity helps differentiate viper bites from cobra envenomation. In our case, faint bite marks without a local reaction were present, but the absence of a witnessed event contributed to a 24-hour delay in diagnosis. The presentation with acute flaccid paralysis and hyperglycemia shifted the initial focus toward GBS and DKA, conditions more commonly encountered in pediatric emergency practice. The bite-to-needle time was 24 hours. Earlier administration of ASV may have reduced the duration of ventilation and hospital stay. Although no commercially available ELISA-based snake venom detection kits exist, secretory phospholipase A2 (PLA2) is a potential biomarker in suspected snakebite cases [11].

Early premonitory symptoms of krait envenomation include abdominal pain, vomiting, excessive sweating, and salivation due to autonomic dysfunction. These are typically followed by a classical descending paralysis, initially involving small muscle groups, resulting in ptosis, external ophthalmoplegia, dysphasia, and nasal twang from bulbar weakness; limb weakness with hypotonia and areflexia; neck flexor weakness (“broken neck sign”); locked-in syndrome; and diaphragmatic involvement leading to respiratory failure in approximately 75% of cases [4,8,12]. Severe envenomation may cause hypertension, hypotension, dilated fixed pupils, and loss of brainstem and spinal reflexes, mimicking brain death [8]. Our patient demonstrated autonomic symptoms (abdominal pain, vomiting, hypotension, dilated fixed pupils), coma, and paralysis. This case highlights that krait bites can present with brain death-like features, and awareness of this can prompt physicians to continue treatment rather than prematurely considering withdrawal of care, as these features are reversible.

Neurotoxins do not cross the blood-brain barrier. Encephalopathy (drowsiness, stupor, or coma) and seizures in snakebite are usually secondary to hypoxia, hypercarbia, or ischemia resulting from respiratory or cardiac failure. Normal neuroimaging in our patient excluded primary neurological disease, although snakebite can sometimes produce abnormal findings such as intracranial hemorrhage or posterior reversible encephalopathy syndrome related to autonomic dysfunction [13].

Most unwitnessed krait bites occur in previously healthy children during the monsoon season, typically presenting in the early morning with acute paralysis, known as EMNS [12,14]. If the bite is unwitnessed, misdiagnosis as hypokalemic periodic paralysis, demyelinating disorders or GBS, acute encephalitis, stroke, head injury, poisoning, DKA, or AIP is common [14]. Our patient presented with EMNS and a brain death-like clinical picture. Empirical ASV in EMNS can be lifesaving when history, examination, and investigations remain inconclusive [15]. Although ASV reactions (anaphylaxis, pyrogenic reactions, and serum sickness-like reactions) are relatively common (5.6%-50%), they can be managed with monitoring, adrenaline, antihistamines, and corticosteroids. Current Indian guidelines recommend administering 10 vials of polyvalent ASV and repeating the dose after six hours if neurotoxic or cardiovascular signs persist or worsen [16,17]. Response to ASV in krait bites is often slow, unlike cobra bites where rapid improvement (restoration of blood pressure, cardiac rhythm, and reversal of weakness) may occur within 30 minutes. This delayed response makes monitoring difficult and often results in higher ASV dosing. Our patient required 30 vials.

Krait venom predominantly contains β-bungarotoxin, which acts mainly at the presynaptic membrane and to a lesser extent at postsynaptic receptors. β-bungarotoxin induces depletion of synaptic vesicles and subsequent degeneration of motor nerve terminals, resulting in severe ASV-resistant paralysis and prolonged recovery [13]. It also depolarizes postsynaptic nicotinic receptors, causing persistent depolarization and making the receptors resistant to reversal by acetylcholinesterase inhibitors. The WHO recommends a trial of acetylcholinesterase inhibitors in all neurotoxic envenomation [16], but their role in krait bites is controversial and often ineffective, sometimes causing paradoxical worsening [13,18,19]. They are most effective for postsynaptic neurotoxins, such as those in cobra venom. Acetylcholinesterase inhibitors were ineffective in our case as well, despite being administered on day 2 for suspected krait envenomation. Mehta et al. reported two cases of krait envenomation in adolescents requiring prolonged ventilation for 15 and 16 days, respectively [20]. Our patient required 22 days of ventilatory support, representing one of the longest durations reported in neurotoxic snakebite.

## Conclusions

CNS involvement in snakebite is typically secondary to metabolic encephalopathy, as neurotoxins do not cross the blood-brain barrier. Brain death-like signs can mislead clinicians, especially when neuroimaging is normal, and may discourage continued treatment. These features are often the result of autonomic dysfunction and do not necessarily indicate a poor prognosis. Neurotoxic envenomation may cause prolonged paralysis and require ventilation for several weeks, but recovery is usually complete. Krait bite should be suspected in previously healthy children presenting with EMNS or a brain death-like presentation during the monsoon season. Krait bites often show subtle or absent fang marks, no local reaction, and there is no routine biomarker to confirm the diagnosis. Empirical ASV should be initiated in such cases when no other clear alternative diagnosis is present.
